# Development and performance evaluation of an improved electric baking oven for baked products

**DOI:** 10.1002/fsn3.3287

**Published:** 2023-03-17

**Authors:** Masud Rana, Sabina Yasmin, Md. Sultan Mahomud, Fatehatun Noor, Md. Sazzat Hossain Sarker

**Affiliations:** ^1^ Department of Food Engineering and Technology Hajee Mohammad Danesh Science and Technology University Dinajpur Bangladesh; ^2^ Department of Food Science and Nutrition Hajee Mohammad Danesh Science and Technology University Dinajpur Bangladesh

**Keywords:** baking oven, biscuit, cake, design, quality

## Abstract

An improved electric baking oven was designed and fabricated using locally available materials for baking cakes and biscuits. Provisions of necessary adjustments were employed for ensuring uniform distribution of heat in all trays of the baking chamber. Its baking characteristics in terms of baking time, specific volume, and product quality in terms of sensory attributes were evaluated. The oven was found to be quite satisfactory in functioning for baking cakes and biscuits. Total time was only 15–28 min for baking the cake samples in the oven. On the other hand, comparatively, a bit longer time 18–35 min required for baking the biscuit samples. Baking cost was lesser in baking small‐sized cakes and biscuits than those of large sized. The quality of baked products was better in terms of taste, color, flavor, texture, and appearance than ordinary market products. Loaf volume of each cake (with 4 × 5 × 8 cm^3^) was 100%, which gave specific volume of 652.8 cm^3^/kg. Similarly, the specific volume of biscuits was 810 cm^3^/kg. The electric baking oven is quite efficient in baking quality cakes and biscuits uniformly, which can be provided to rural small entrepreneurs for commercial manufacturing of biscuits and cakes.

## Graphical abstract

An improved electrical baking oven was designed for baking bakery products. The electric baking oven is quite efficient in baking quality cakes and biscuits uniformly, which can be provided to rural small entrepreneurs for commercial manufacturing of biscuits and cakes.

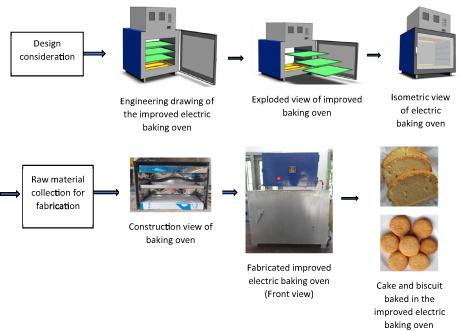

## 1 INTRODUCTION

Baking is a method of preparing food by using heat generally in an oven, but can also be done in another way like using a cooker. It is a universal cooking method of heating food inside an oven at a uniform temperature. In this process, heat is transferred to the product load mainly by means of radiation and convection. Although it is a widely known phenomenon, complex thermal, chemical, and mass transfer process occurs within product, and thus its properties change during the process (Purlis, 2012). The most commonly baked items are cakes and biscuits. At present, baking product is the most important part of breakfasts and snacks. There are a large number of baking industries all over the world. The art of baking remains a fundamental skill and is important for nutrition. Over the years, improvements have been made in electric baking ovens and this trend still continues. This has led to the incorporation of features like a thermostat which turns the oven on and off and also helps in regulating the temperature of the oven.

An oven is a thermally insulated chamber used for the heating, baking, or drying of a substance. The earliest ovens were found in central Europe and dated to 29,000 BC, those were used as roasting and boiling pits located within yurts structures (Srinivasan et al., 2014). Ovens have been used since prehistoric times by cultures that lived in Egypt. Hence, before the intervention of modern baking ovens, people had alternative means of cooking and baking. The different types of baking ovens are earth oven, ceramic oven, gas oven, wall oven, and electric oven. By considering their energy source, ovens can be broadly classified into two groups, fuel‐based and electric ovens (Mullinger & Jenkings, 2008). Electric resistance heating has various advantages over systems based on fuel combustion, such as increased control accuracy and heating rate. Researchers have analyzed diverse types of ovens (Khatir et al., 2012; Mirade et al., 2004). Development of a small‐scale electric oven for baking quality products is important to create business opportunities for an entrepreneur. The insulator helps in preventing heat loss from the oven and may allow the baking oven to be turned ON and OFF automatically at preset times, and it can also be used to shut the baking oven off when the food is completely cooked or when the cake is completely baked to the desired degree.

Several works have been carried out on baking ovens due to its importance and ease of operation. Genitha et al. (2014) developed a domestic gas oven by which higher energy efficiency was obtained by reducing the energy, cost, and time of baking. Adegbola et al. (2012) developed a low‐cost domestic electric baking oven with the incorporation of a blower for improved efficiency. Boulet et al. (2010) designed a bakery pilot oven by CFD modeling. Therdthai et al. (2003) designed an efficient and cheap gas oven by using the software 3D solid works which showed the pictorial views, lines, and dimensions for the fabrication. In the fabrication process, the major design considerations were considered as emphasized by other researchers (Akinnuli & Olufemi, 2019). However, it should be noted that the existing baking oven takes much time because of the long waiting time of the users to have their food ready. Also, the performance of those ovens is very low and not up to standard. Hence, to improve the performance of the existing oven, there is a need to develop an improved electric baking oven through the incorporation of thermostat and interlock switch. The proposed electric baking oven would be portable, readily available, relatively cheaper, and easy to install and operate including high durability compared to other types of ovens. This oven is also aimed to develop targeting daily local market demand for an area so that small entrepreneurship can be encouraged. The performances of the oven in terms of baking characteristics and quality of baked products have been evaluated.

## 2 MATERIALS AND METHODS

### 2.1 Collection of materials

Required materials such as mild steel sheet grade 1, stainless steel sheet grade 1, electric cable, temperature sensor, and conductor were purchased from the local market of Dinajpur. Glass wool insulator (grade 1) and electric heater were purchased from Nowabpur, Dhaka.

### 2.2 Design procedure of the improved electric baking oven

A comprehensive approach was used for designing and fabricating the baking oven to be used for baking various products. Locally available common materials (mild steel sheet, stainless steel sheet, glass wool, and electric wire) were used for fabricating the oven (Table 1).

**TABLE 1:** Construction materials used for the improved electric oven.

S/N	Name of the components	Materials
1	Outer part	Mild steel grade 1
2	Inner part	Stainless steel grade 1
3	Insulator	Glass wool grade 1
4	Control box	Mild steel
5	Tray	Stainless steel
6	Nut–bolt	Cast iron
7	Oven lock	Mild steel
8	Air hole	Mild steel
10	Oven stand	Angle (Mild steel 1.5, 3 mm)
11	Door handle	Iron rod
12	Electrical wire	Copper cable (4 mm)

### 2.3 Design consideration

During the development stage of the electric oven, a few important design considerations were taken into consideration to customize the size and volume of the electric oven to make it capable of baking various types of cakes and biscuits such as: 

1. The oven can be fabricated in local workshops by local mechanics.
2. It will be energy efficient. It will be compact in size and attractive in shape.
3. The oven can be operated at variable temperatures within 150–250°C.
4. The fabrication cost is reasonable (<US$200).
5. The loading and unloading system will be manual.
6. Finally, it will be operated by a single‐phase electric line for operating in rural areas.

### 2.4 Selection of construction materials for the oven

The following materials were selected on the basis of the design consideration and requirements for fabrication of the electric baking oven.

### 2.5 Design calculation of various parts of the oven

Selection of suitable dimensions of the different components of the oven such as housing unit, baking chamber, control box, oven tray, and heating coil was determined. All the dimensions of the different components were finalized to make the oven attractive to look at and easy to operate with transportation facility.

### 2.6 Design of the main structure

The main structure represents the entire outlook of the electric oven (Table 2). The dimension of housing unit is selected as 60 × 40 × 45 cm (length × width × height) and easy operation to make it attractive in appearance. The housing unit of the electric oven is made of 16‐gauge mild steel sheet, which makes the oven stronger and have smooth surface finish. The wall of the housing unit was insulated by glass wool maintaining 25.4 mm (1 inch) on all sides.

**TABLE 2:** Details of the materials used in the fabrication steps of the electric baking oven.

S/N	Operation	Descriptions	Tools
1	Material collection	Required materials for the construction were purchased from Dinajpur and Dhaka.	Free hand
2	Dimension of parts	The overall dimensions of the oven were 60 cm × 40 cm × 45 cm. To maintain selected size, proper measurement of different parts of the electric oven was ensured.	Steel tape, Scriber meter rule, etc.
3	Cutting of different parts	Proper cutting of different parts was performed according to the required dimension. The dimensions of baking chamber were 55 cm × 35 cm × 40 cm. Therefore, tray size was 55 cm × 35 cm.	Plate cutting machine and hammer.
4	Drilling (hand drilling)	Drilling of hole in different parts of the oven was done for different purposes such as joining, keeping air holes, setting the temperature meter inside the wall of the oven, etc.	MS drill bit and SS drill bit were used
5	Bending	Parts were bent to give required shape for different purposes also done in local workshop.	Plate bending machine
6	Welding and repeating	Adequate welding of the oven in different sections and joining different parts to each other unit were properly done.	Electrical welding machine and repeating machine
7	Insulation	Providing grade 1 glass wool into the oven walls as shown in the AutoCAD design was done manually.	Free hand
8	Electrical connectivity	The electrical wire connection from the electrical component to the control box and the heating coil at the outside of the oven was completed by the local electrician.	Free hand
9	Painting	Painting of the exterior view of the body of the baking oven was done by an experienced painter.	Painting brush

### 2.7 Design of the baking chamber

The baking chamber is an air‐tight chamber where the heating process is carried out (Table 2). The baking chamber of the oven was placed inside the housing unit. The dimension of the baking chamber is selected as 55 × 35 × 40 cm (length × width × height). Stainless steel sheet of 1 mm thickness was used for fabrication purposes of baking chamber to make the oven food grade and to minimize heat loss as well. The baking chamber is equipped with two electric heaters of 1000 W power each, one at the top and another at bottom of the chamber. Two trays and temperature sensors are arranged in the middle of the baking chamber.

### 2.8 Design of the control box

The control box contains the control panel instrument of the electric oven. The control box of the electric oven is made of mild steel sheet of 16‐gauge thickness having the dimension of 30 × 20 × 24 cm (length × width × height). It contains 60 conductors, three magnetic circuit breakers, temperature meter, timer, and light indicator. Temperature meter and light indicator were set outside the wall of the oven and other parts are placed inside the box.

### 2.9 Design of the oven tray

The dimension of the tray was selected as 55 × 35 cm (length × width) to accommodate inside the baking chamber (Table 2). Two trays are provided in the oven cavity; both trays are made of stainless steel sheets of 1 mm thickness. The oven works by the convection process that enhances the movement of the hot air to the upper and lower tray of the oven eventually. The distance from upper tray to the lower tray was kept at 10 cm.

### 2.10 Selection of the heating coil

The bimetallic thermometer ranging the temperature from 0 to 300°C was placed in the side wall of the control box. The thermometer sensor will be placed in the center portion of the baking chamber to ensure that the thermometer should detect the temperature of both the lower and the upper layer of the electric oven.

### 2.11 Fabrication of the electric baking oven

**FIGURE 1:**
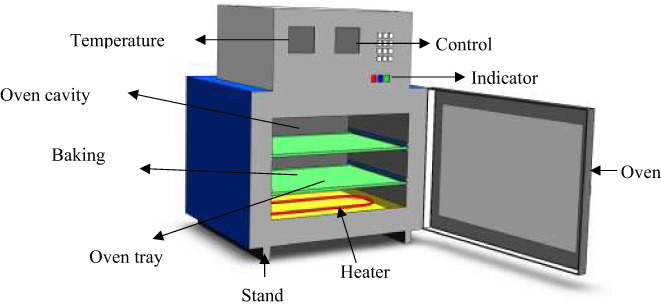
Design drawing (pictorial view) of the electric baking oven. Engineering drawing of the electric baking oven. Detailed engineering drawings including 3D views are illustrated in this figure.

The fabrication and assembly works of different parts of the electric oven were carried out using the data obtained from the above computations (Figure 1). All the parts of the oven such as housing unit, baking chamber, perforated covers for heat distribution, heating coils, and temperature sensors were fabricated using locally available materials in a local workshop with the help of local experienced technicians. Other parts of the oven were set up at different points according to requirements. The heat from the heater heated the air inside the oven for baking. The density of the air decreases as the air gets heated. This heated air removes moisture from product and passes through the air hole causing uniform baking of the product.

### 2.12 Performance calculation of the improved electric baking oven

#### 2.12.1 Calculation of electric energy consumption (kWh/kg)

The following formula (Equation 1) was used for calculating the electrical energy actually consumed by the electric heater of the oven. Voltage and current were recorded approximately at 10 min intervals.

(1) $$E_{\text{heater}}=\frac{\left(\mathrm{VI}\;\mathrm{Cos}\;Ѳ\times t\right)}{1000}$$

where *V* = line voltage (volt); *I* = line current (amp); Cos Ѳ = power factor; and *t* = actual heater operating time (h).

Finally, the specific energy consumption was calculated as total energy/total product in a batch.

#### 2.12.2 Calculation of specific thermal energy required for baking of products

The specific thermal energy required for baking was calculated according to the equation.

(2) $$\mathrm{Es}=m_{b}\times C_{b}\times \left(T-T_{0}\right)\times t$$

where average baking (oven) temperature = *T*, average mass of a cake/biscuit = *m*_b_, specific heat capacity of cake/biscuit = *C*_b_, ambient temperature = *T*_0_, and total baking time = *t*.

### 2.13 Preparation of baked products

#### 2.13.1 Biscuit preparation process

Whole‐grain wheat flour of the Pusti brand was collected from a local vendor, Dinajpur (Table 3). The moisture and protein content of the flour was 10% and 12%, respectively. The previously sieved flour was taken into a bowl and baking powder was added to the flour. Butter was cut into pieces and flour was added to it. After that, flour and butter were rubbed until the mixture is crumbling. Powder sugar and milk powder which contain 24% protein and 39% fat were added at a time and mixed properly to make smooth dough holding for 15–20 min for proper aeration. Dough was rolled in a wooden chapatti roller and wheat flour was dusted into the batter. Then, the dough was cut into desired round shape using biscuits mold. Finally, biscuits were placed on oiled tray and baked at 200–210°C for 20–25 min. After baking, the tray was removed from the oven and cooled on a rack for 20–25 min.

**TABLE 3:** Ingredients for making biscuits.

Serial No.	Ingredients name	Amount/quantity (g)
1	Flour	600
2	Sugar	200
3	Butter	150
4	Baking powder	10
5	Milk powder	40

#### 2.13.2 Cake preparation process

Whole‐grain wheat flour along with all necessary ingredients was collected from a local market, Dinajpur, for making cakes (Table 4). After that, about 88 g egg was taken into a bowl and mixed well with a mixer for 3 min. After mixing of egg, sugar, butter, and milk powder were added and mixed well. Therefore, flour of Pusti brand and baking powder were added to the batter and mixed properly. No water was added to the batter. Finally, the mixture was poured into a plain cake pan and baked at 170–180°C for about 25–30 min. The baked product was removed from the oven and cooled on a rack for 15–20 min at ambient temperature.

**TABLE 4:** Ingredients for making plain cakes.

Serial No.	Name of the ingredients	Amount/quantity (g)
1	Flour	500
2	Egg	88
3	Sugar	250
4	Butter	130
5	Baking powder	20
6	Milk powder	50

### 2.14 Specific volume of cakes and biscuits

Volume of cakes and biscuits was determined with rapeseed replacement method and the specific volume of cakes and biscuits was calculated as the ratio of volume to weight (cm^3^/g) according to the method described in AACC (2002).

### 2.15 Sensory attributes of baked product

Sensory analysis (color, flavor, taste, texture, and overall acceptability) of cakes and biscuits was evaluated by 30 semitrained panelists using a 9‐point hedonic rating scale according to Lawless and Heymann (2010). Randomly coded samples were presented to 30 semitrained panelists and asked to rate the color, flavor, texture, and overall acceptability on a 1–9 point scale, where 1 = dislike extremely, 2 = dislike very much, 3 = dislike moderately, 4 = dislike slightly, 5 = neither like nor dislike, 6 = like slightly, 7 = like moderately, 8 = like very much, and 9 = like extremely.

### 2.16 Statistical analysis

The obtained data from sensory attributes were expressed as mean ± standard deviation. Data were analyzed by SAS (Version 9.3) statistical software. One‐way analysis of variance was done using ANOVA procedures. Significant differences among the means of response values were determined by Duncan's multiple‐range test (DMRT) at the 95% confidence level.

## 3 RESULTS AND DISCUSSION

### 3.1 Design and fabrication success of the electric baking oven

**FIGURE 2:**
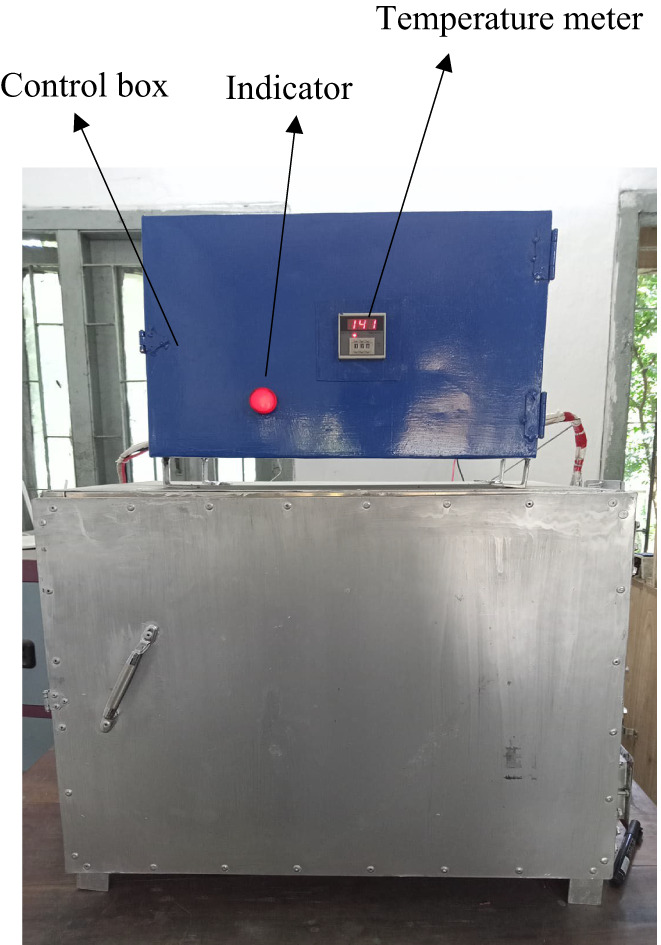
Front view of the electric baking oven.

**FIGURE 3:**
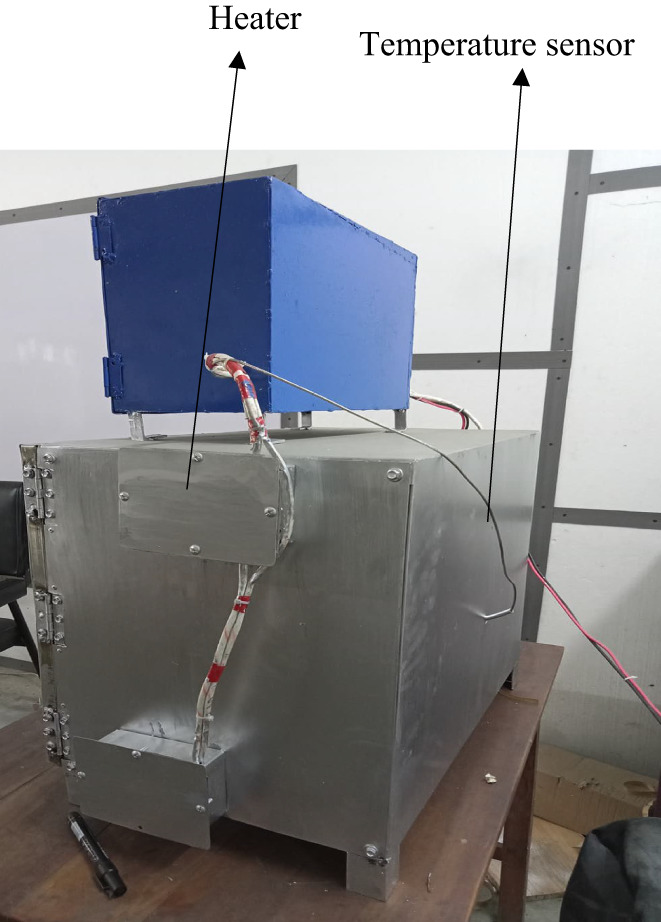
Rear view of the electric baking oven.

An electric oven was successfully designed and fabricated as per estimated specifications. The photographic views of the developed electric oven are shown in Figures 2 and 3. The oven was finally installed in the Research Laboratory of the Department of Food Engineering at Hajee Mohammad Danesh Science and Technology University (HSTU), Dinajpur. The oven was operated successfully for baking numerous types of baked products.

### 3.2 Baking characteristics of the electric baking oven

The electric oven was found to bake food by heating elements placed in the oven after being heated to the desired temperature. Different types of products were baked in the electric oven. A total time 15–28 min was required for baking of cake and 18–35 min was required for baking of biscuits based on the shape and size of cakes and biscuit. It took lesser time thus found to be less expensive than gas ovens. It can bake food evenly and quite easily. It is equipped with buttons that need to be pressed to achieve the desired set temperature. It is also easy to clean and maintain. Loaf volume of each cake (with 4 × 5 × 8 cm^3^) was 100%, which gave specific volume of 652.8 cm^3^/kg. Similarly, the specific volume of biscuit was 810 cm^3^/kg. The results are in agreement with (Aly & Seleem, 2015; Jennifer Coleman et al., 2013).

### 3.3 Performance analysis of electric baking oven

The performance of the electric oven was analyzed according to different parameters such as obtained desired level of temperature, time to raise baking temperature, and consumption of energy during baking which has been in the following section.

### 3.4 Evaluation of temperature in the oven during baking

**FIGURE 4:**
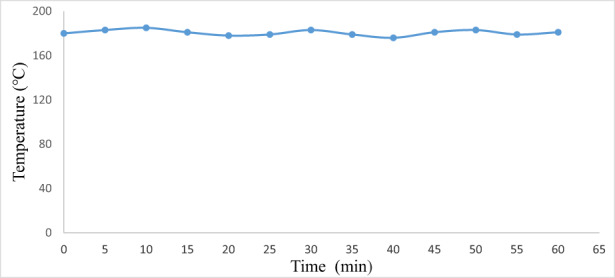
Evaluation of temperature in the oven during baking.

The electric baking oven was tested in order to determine the maximum temperature generated in the oven and for the calibration of the thermostat. After assembling the oven, the first experiment was done with it using a thermometer and a timer to discover the effective heating of the oven. A certain temperature is fixed such as 180°C and temperature was recorded in 5 min intervals, and a graph was plotted with time and temperature, where the *X* axis represents time and the *Y* axis represents temperature (Figure 4). The graph showed the fluctuation of temperature to be about ±5°C and higher temperature was recorded at 185°C and lower temperature was at 176°C, which indicated better performance than other electric ovens as reported by Olugbade and Ojo (2018) and Ologunye et al. (2020).

### 3.5 Temperature raises experiment in the oven

**FIGURE 5:**
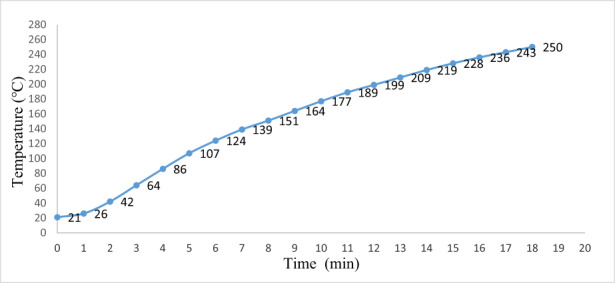
Measurement of time required to raise the temperature in the oven.

The oven was heated with electric energy and temperature was recorded at every 1 min interval. The recorded value was plotted into a graph where the *X* axis represents time and the *Y* axis represents temperature. From Figure 5, it has been shown that the oven heating coil reached its maximum heating temperature in 18 min which is 250°C.

### 3.6 Measurement of time required to cool the oven

**FIGURE 6:**
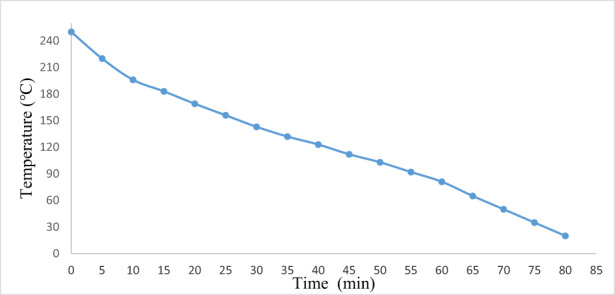
Measurement of time required to cool oven from 250 to 20°C.

The oven was heated with electric energy until its temperature stabilized. Then, electric energy was disconnected and the fall in temperature of the electric oven was recorded. The results of fall in temperature are shown in Figure 6. It has been seen that the temperature fall evolution of the oven with time.

### 3.7 Electric energy consumption of the oven

**FIGURE 7:**
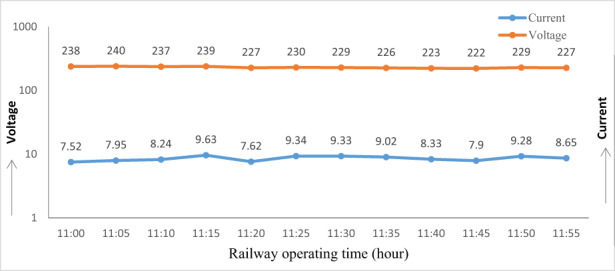
Measurement of current and voltage in the oven during operating time.

Current and voltage were recorded when the oven was running and a graph was plotted which showed the relationship between current and voltage at different times as shown in Figure 7; we found maximum current as 9.63 A and voltage of 240 V.

### 3.8 Specific electric energy consumption of the oven

During the period of baking, electric energy was converted to heat and used to remove moisture from baked products. For calculation of unit cost of baking, it is important to determine the energy consumption of the oven (Table 5). So, for calculation, we measure current and voltage of the oven at different times, and calculated the average value of current as 8.57 A and voltage 230 V. The specific electric energy consumption for baking biscuits was 0.53 kWh/kg, whereas the specific electric energy consumption for baking cake was 0.88 kWh/kg. More or almost double specific energy consumption value was found to be utilized in baking similar products (Ologunye et al., 2020; Olugbade & Ojo, 2018; Paton et al., 2013).

**TABLE 5:** Energy consumption pattern of the oven in load condition at constant baking temperature (°C).

ON time	OFF time	Operating duration (s)
15:46:02	15:47:34	92
15:51:05	15:52:40	95
15:56:42	15:57:55	73
16:02:12	16:03:35	83
16:07:15	16:08:42	87
16:13:20	16:14:29	69
16:18:35	16:19:45	70
16:23:56	16:25:12	68
16:29:27	16:30:36	69
16:34:45	16:36:02	77
16:39:55	16:41:06	71
16:45:06	16:46:22	76

Besides, temperature controller was used to control temperature inside the oven at a certain range. Therefore, temperature was set at 180°C for baking cakes; it turns OFF the heater when temperature reached the desired level. After a certain period, temperature varies at 180–185°C and then gradually starts to decrease. When the temperature reached 178°C, again heater was turned ON and produced heat until it reached 180°C. For calculation of energy consumption, time was recorded during it turned OFF and ON because in turned OFF time, no energy was used by the oven. During calculation of unit cost per hour in the oven, it is important to know how much time the heater of the oven was running. Table 5 shows the recorded data for calculation of unit cost per hour. From Table 5, it has been seen that total running time of the oven in 1 h was 15.5 min. So, using Equation 2, we calculate the specific electric energy consumed in 15.5 min, when the oven running at 1 h was 0.59 KWh.

### 3.9 Baking performance of electric baking oven

#### 3.9.1 Baking time for different products

Different types of products were baked in the electric oven such as cakes and biscuits. The time required to bake cakes and biscuits depended on size and shape of the products. Generally, larger‐size cakes required much more time than smaller ones. The time required to bake different sizes of plain cakes and round biscuits is shown in Table 6.

**TABLE 6:** Time required for baking different size cakes and biscuits. (a) For cakes; (b) For biscuits.

(a)			
Cake size	Weight of cake (g)	Required time (min)	Temperature (°C)
Small size	40–45	18–20	170–180
Medium size	100–110	22–25	
Larger size	190–200	30–35	

(b)			
Biscuit size	Weight of biscuit (g)	Required time (min)	Temperature (°C)
Small size	10–12	15–18	200–210
Medium size	20–22	20–23	
Larger size	30–32	25–28	

#### 3.9.2 Uniform baking behavior of cake in the oven

**FIGURE 8:**
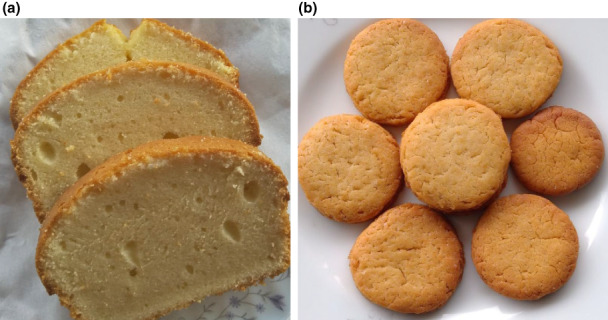
(a) Baked plain cakes in the improved electric oven. (b) Baked round biscuits with attractive color and texture.

In the electric oven, baking plain cake has a uniform result. Time required for baking cakes was found to be varying from 18 to 35 min and the temperature varied from 170–180°C. Heats were uniformly distributed inside the oven which resulted in well baking of the cake samples. Color, flavor, and texture of cakes were acceptable and attractive. Some illustrations of baked cakes are shown in Figure 8a.

#### 3.9.3 Uniform baking behavior of biscuits in the oven

In the electric oven, biscuits were baked at different times and temperatures. The time required for baking biscuits varied from 15 to 28 min and temperatures varied from 200 to 210°C. Heat was uniformly distributed inside the oven, resulting in well‐baked biscuits. Color, flavor, and texture of biscuits were also acceptable. Brunet et al. (2011) reported that baking time for cookies and biscuits ranged from 2.5 to 15 min. Some illustrations of baked round biscuits are shown in Figure 8b.

#### 3.9.4 Sensory evaluation of baked product

Sensory attributes of cakes and biscuits baked in an improved baking oven are shown in Table 7a,b. It is seen that there was no significant difference between commercial cakes and improved baking oven cakes. On the other hand, a significant difference was observed between commercial biscuits and biscuits baked in the improved oven. This may be due to mixing ingredients difference during making batter (Figoni, 2011).

**TABLE 7:** (a) Sensory attributes of cake baked in the improved baking oven. (b) Sensory attributes of biscuits baked in the improved baking oven.

(a)		
	Commercial cakes	Improved baking oven cakes
Color	8.33^a^ ± 1.07	8.42^a^ ± 0.67
Flavor	8.08^a^ ± 0.90	8.08^a^ ± 0.67
Taste	7.67^a^ ± 1.44	8.17^a^ ± 0.58
Texture	8.08^a^ ± 1.16	7.33^a^ ± 0.78
Overall acceptability	7.75^a^ ± 1.22	8.08^a^ ± 0.51

(b)		
Sensory attributes	Commercial biscuit	Improved baking oven biscuit
Color	8.7^a^ ± 0.48	7.8^b^ ± 0.42
Flavor	8.5^a^ ± 0.71	7.6^b^ ± 0.97
Taste	8.7^a^ ± 0.48	7.6^b^ ± 0.97
Texture	8.5^a^ ± 0.52	6.0^b^ ± 1.63
Overall acceptability	8.5^a^ ± 0.71	7.1^b^ ± 0.73

*Note*: Superscript letters indicate significant difference among the sensory attributes of cakes.

## 4 CONCLUSIONS

An electric baking oven was designed and fabricated with locally available materials from a local workshop by local technicians. Mild steel sheet and stainless steel sheet were used for the construction of the outside and inside of the body, respectively, of the oven. Glass wool which has thermal conductivity of 0.04 (w/m K) was used as the insulating material. A temperature control device was also used to ensure that the oven temperature can be regulated properly. The oven was constructed for home use and small entrepreneur because of its small size. The simplicity of the oven makes it unique with various advantages such as easy to fabricate, easy to clean, easy to disassemble and assemble, low cost for a family of average income, portable, allowing fast baking time, and making the cooking of conventional foods efficient. There was no significant difference between sensory attributes of commercial cakes and improved baking oven cakes. On contrary, significant difference was observed in biscuits. Therefore, the proposed oven can be one of the most suitable electric baking ovens for baking cakes and biscuits. However, this research has an opportunity for further modification, development, and fabrication of electric baking ovens for small household units and at a scale for the production of quality baked product.

## FUNDING INFORMATION

The authors received financial support from the Institute of Research and Training (IRT), HSTU, in the year 2019–2020 (Project No. 84) for this study.

## CONFLICT OF INTEREST STATEMENT

The authors declare no conflict of interest.

## ETHICAL APPROVAL

Ethics approval was not required for this study because this is not a medical study nor any nutritional trial was conducted on humans.

## Data Availability

The data supporting the findings of this research will be available on request.
